# Diagnosis of Tuberculosis by Using a Nucleic Acid Amplification Test in an Urban Population with High HIV Prevalence in the United States

**DOI:** 10.1371/journal.pone.0107552

**Published:** 2014-10-23

**Authors:** Miwako Kobayashi, Susan M. Ray, John Hanfelt, Yun F. Wang

**Affiliations:** 1 Department of Medicine, Emory University School of Medicine, Atlanta, Georgia, United States of America; 2 Department of Pathology and Laboratory Medicine, Emory University School of Medicine, Atlanta, Georgia, United States of America; 3 Department of Biostatistics, Emory University Rollins School of Public Health, Atlanta, Georgia, United States of America; 4 Grady Memorial Hospital, Atlanta, Georgia, United States of America; Universidad Nacional de La Plata., Argentina

## Abstract

**Background:**

Use of nucleic acid amplification tests (NAAT) for the diagnosis of *Mycobacterium tuberculosis* (TB) has been recommended on respiratory specimens submitted for acid-fast bacilli (AFB) testing. It also helps distinguish between TB and non-tuberculous mycobacteria (NTM) species in a setting where NTM rates are relatively high. The purposes of this study are to describe the trend and characteristics of all AFB smear-positive respiratory samples that underwent amplified Mycobacterium tuberculosis direct (MTD) testing, a type of NAAT, and to evaluate the clinical utility and necessity of the test for diagnosis of TB in a population with high-HIV prevalence.

**Methods:**

Prospective diagnostic testing and retrospective data analyses were conducted on all AFB smear-positive respiratory samples that underwent MTD testing from 2001 to 2011 at Grady Memorial Hospital (GMH), Atlanta, USA. The test performance was compared to culture.

**Results:**

A total of 2,240 AFB smear-positive specimens from 1,412 patients were tested and analyzed in the study. The proportion of specimens that were culture-positive for TB was 28.5%. Sensitivity, specificity, positive predictive value, and negative predictive value of the MTD were 99.0%, 98.0%, 95.3% and 99.6%, respectively. A downward trend was observed in the yearly numbers as well as the proportions of MTD-positive specimens during the study period (p<0.01). There were 2,027 (90.5%) specimens from patients with known HIV status, of which 70.6% was HIV positive and the majority of them (81.8%) had CD4 counts of less than 200 cells/µL. HIV-positives were more likely to have NTM compared to HIV negatives (67.7% vs. 35.4%, p<0.01).

**Conclusion:**

Despite the decrease in the incidence of TB, NAAT continues to be an accurate and important diagnostic test in a population with high HIV prevalence, and it differentiates TB and NTM organisms.

## Introduction

Use of nucleic acid amplification tests (NAAT) for the diagnosis of *Mycobacterium tuberculosis* (TB) has allowed rapid identification and diagnosis of TB. The US Centers for Disease Control and Prevention (CDC) published the first guideline in 1996, then an updated guideline in 2009 [1] and suggested that “*NAA should be performed on at least one respiratory specimen from each patient with signs and symptoms of pulmonary TB for whom diagnosis of TB is being considered but has not yet been established*”. Compared with acid-fast bacilli (AFB) smear microscopy, the addition of NAAT provides better positive predictive value with AFB smear-positive specimens [1] and its usefulness has also been reported in smear-negative specimens [1], [2]. In addition, NAAT provides quicker results compared to confirmation by culture growth, a process that could take weeks.

Grady Memorial Hospital (GMH) is a 960-bed urban teaching hospital in metro Atlanta. GMH primarily serves the residents in two counties (Fulton and DeKalb) which have the highest TB incidences in Georgia [3]. In 1992, in response to a nosocomial outbreak of pulmonary TB, GMH implemented an enhanced isolation policy [4]. The policy requires airborne isolation of all patients with active TB, those admitted with TB in their differential diagnoses, patients for whom AFB sputum smears and cultures ordered, and patients with HIV infection presenting with respiratory symptoms and/or abnormal chest radiograph [5]. For patients who remain hospitalized, isolation is continued until AFB smears are reported as negative and an alternate explanation for the presenting illness has been established, or when AFB smears are negative and the patient has completed at least two weeks of therapy for suspected TB.

The GMH microbiology laboratory implemented in 2000 the FDA-approved Amplified Mycobacterium tuberculosis Direct Test (MTD), a type of NAAT from Hologic Gen-Probe (San Diego, CA), for AFB smear-positive respiratory specimens (first specimen only) following the CDC guidelines [1], [6]. After MTD was introduced, an additional criteria used to stop airborne isolation was a negative MTD result from a smear positive respiratory specimen. The test manufacturer reports high sensitivity (96.9%) and specificity (100%) when this test is used for AFB smear-positive specimens [7], suggesting that it would not only be useful for the rapid diagnosis of TB, but also to distinguish between TB and non-tuberculous mycobacteria (NTM) species. One of the unique features of the patient population seen at GMH is a relatively high HIV prevalence, which is supported by the fact that metro Atlanta has one of the highest HIV incidences among the metropolitan statistical areas of residence [8]. Atypical presentations of pulmonary TB has been described in patients with HIV, especially in those with advanced immune suppression [9]. In addition, NTM species including *Mycobacterium avium* complex (MAC) in particular, is known to frequently colonize lung secretions of HIV-infected individuals [10], and while these organisms are commonly suspected as the cause for AFB smear positive respiratory specimens, reports have also suggested that the presence of NTM may result in false-positive MTD results [11], [12].

The purposes of this study are to describe the trends and characteristics of all AFB smear-positive respiratory samples that underwent MTD testing prospectively from 2000 to 2011 at GMH, and to evaluate the clinical utility of the test for diagnosis of TB in the hospital patients with high-HIV prevalence.

## Methods

### Clinical Specimens

All AFB smear-positive respiratory samples that had MTD testing at GMH from 2001 to 2011 were included in the study. The respiratory specimens included sputa, bronchial washings, and tracheal aspirates.

### Specimen Processing and Culture

The respiratory specimens were first decontaminated with N-acetyl-L-cysteine-sodium hydroxide (NACL), and were concentrated with centrifugation (3000 g for 15 minutes), according to standard procedures [13]. After centrifugation, the supernatant was decanted, and phosphate buffer was added to the pellet. Part of the sediment was used to prepare an AFB fluorochrome smear. Approximately 0.5 ml was used to inoculate into MB/BacT bottle and incubated in the BacT/ALERT 3D system (bioMérieux, Durham), and about 0.25 ml onto a Middlebrook 7H11 plate. The cultures were incubated at 37°C for 5–6 weeks. Isolates of mycobacteria were identified by DNA probes (AccuProbe, Hologic Gen-Probe, San Diego, CA) or by conventional biochemical tests, according to standard protocol [13]. The remaining sediments were stored at 2–8°C for up to 3 days until they were tested for MTD, or at −70°C if they needed to be stored for more than 3 days.

### MTD Test

In principle, the MTD test was only performed on respiratory specimens that were AFB smear-positive. If the first specimen from a patient resulted in an inconclusive test result, MTD was repeated on the second specimen. MTD testing and interpretation of the results were done according to the manufacturer's protocol [14]: relative light units (RLU) of more than 500,000 was considered positive; RLU of less than 30,000 were considered negative; and RLU between 30,000 to 500,000 were considered equivocal. Equivocal results were repeated, and if the repeat testing continued to show equivocal results, the results were considered un-interpretable. The turnaround time, i.e., the duration between specimen collection to the report of MTD test result, was also recorded.

### Patient Information

For each specimen, we collected information on patient demographics (age, gender), and HIV status if available. For those who were HIV positive, CD4 count at the time of MTD testing was also collected. For MTD results that were un-interpretable or were discordant with the mycobacterial culture results, we further reviewed the patients' medical records to obtain additional clinical information.

### Data Analysis

For the purpose of calculating the testing characteristics of MTD, mycobacterial culture results from the same samples for AFB smear and MTD were used as the reference standard. For the MTD results, we used the results that were reported to the clinicians as the final results, as some samples had the test repeated before the results were reported. MTD results that were considered to be un-interpretable were excluded from the calculation of the testing characteristics. Although the laboratory protocol was to perform the MTD test on only the first smear-positive specimen, the test was occasionally performed on subsequent specimens mainly per the clinician's request. Therefore, in addition to reviewing the results of all the specimens individually (“per specimen”), we also looked at the results by “per unique visit” by only including the first MTD test result within a 30-day period per patient if the specimens came from the same patient, and disregarded the results that came from subsequent specimens.

We then calculated the testing characteristics after adjustment (adjusted MTD): if the MTD result was either un-interpretable or negative, but previously had a positive TB sample (within one year), the MTD result was considered positive in the adjusted MTD, as MTD is not recommended on samples from those already on treatment [14]. In addition, if a known TB patient (culture positive within one year) had a negative culture result but a positive MTD, we also considered the sample to be culture-positive in the adjusted MTD analysis.

Lastly, the trends in the numbers of MTD tested smear-positive samples and the proportions of those that had positive results were calculated during the study period. Statistical p-values based on Pearson chi-square tests were used to evaluate the significance of year-to-year changes. For the categories with more than 5 observations, p-values were calculated using two-sample test of proportion. The p-values for the median were calculated using the K-sample median test. The calculations were done by STATA/IC 10.0 (StataCorp, College Station, TX).

### Ethics Statement

The study protocol received approval from the Institutional Review Board at Emory University (IRB00057687).

## Results

### MTD Test Performance

There were a total of 2,240 AFB smear-positive specimens from 1,412 patients that underwent MTD testing prospectively during the period. When the specimens were counted per unique visit, there were 1,644 samples from 1,412 patients. The median turnaround time for the MTD test results was 2 days for both per specimen and per unique visit (range 0–46 for per specimen, 0–40 for per unique visit). The testing characteristics of MTD are summarized in Table 1 (without adjustment) and in Table 2 (after adjustment). The pre-adjusted results (Table 1) were obtained by excluding the 40 samples (1.8%) that had an equivocal result, whereas after adjustment, only two samples (0.09%) were classified as equivocal (Table 2). Sensitivity, specificity, positive predictive value (PPV) and negative predictive value were 99.0%, 98.0%, 95.3% and 99.6% respectively, and the numbers improved slightly after adjustment (99.4%, 98.8%, 97.1%, 99.7% respectively).

**Table 1:** Testing characteristics of MTD without adjustment.

Per sample	MTD result (number of samples)		Total
TB culture result	Negative	Positive	
Negative	1540	31	1571
Positive	6	623	629
Total	1546	654	2200
	Sensitivity/Specificity (%)		99.0/98.0
	PPV/NPV (%)*		95.3/99.6

Note: * PPV = positive predictive value, NPV = negative predictive value.

**Table 2:** Testing characteristics of MTD after adjustment.

Per sample	MTD result (number of samples)		Total
TB culture result	Negative	Positive	
Negative	1571	19	1590
Positive	4	644	648
Total	1575	663	2238
	Sensitivity/Specificity (%)		99.4/98.8
	PPV/NPV (%)*		97.1/99.7

Note: * PPV = positive predictive value, NPV = negative predictive value.

Among all 2,240 smear-positive respiratory specimens (per specimen), there were 34 (1.5%) that had MTD results that were discordant from the AFB culture: 28 (1.3%) had a false-positive MTD result and 6 (0.3%) had a false-negative MTD result (data not shown). Of the 28 false-positives, 9 either previously (within one year) had TB or had TB isolated from other specimens. Of the 6 false-negatives, 2 samples had mixed culture results (TB and MAC). Additionally, there were 40 (1.8%) specimens that initially had an equivocal MTD result. 36 of the 40 (90%) had repeat MTD testing: 5 were positive (1 false-positive), 28 were negative (2 false-negatives), and 3 had repeat results that were also equivocal.

### Trends of MTD Testing During the Study Period

**Figure 1:**
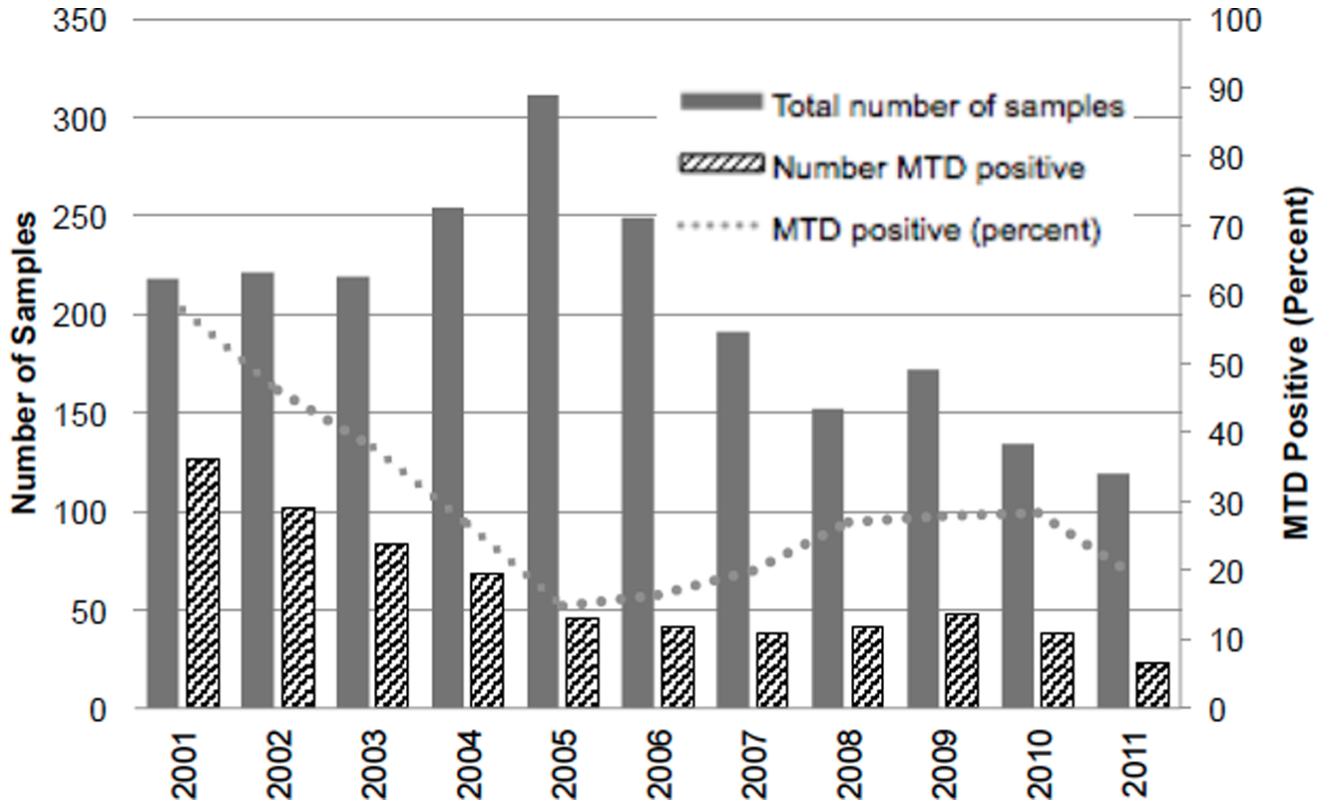
Trend in the number of samples submitted for MTD testing, number with positive MTD, and the percent of samples with positive MTD by year, per specimen.

**Figure 2:**
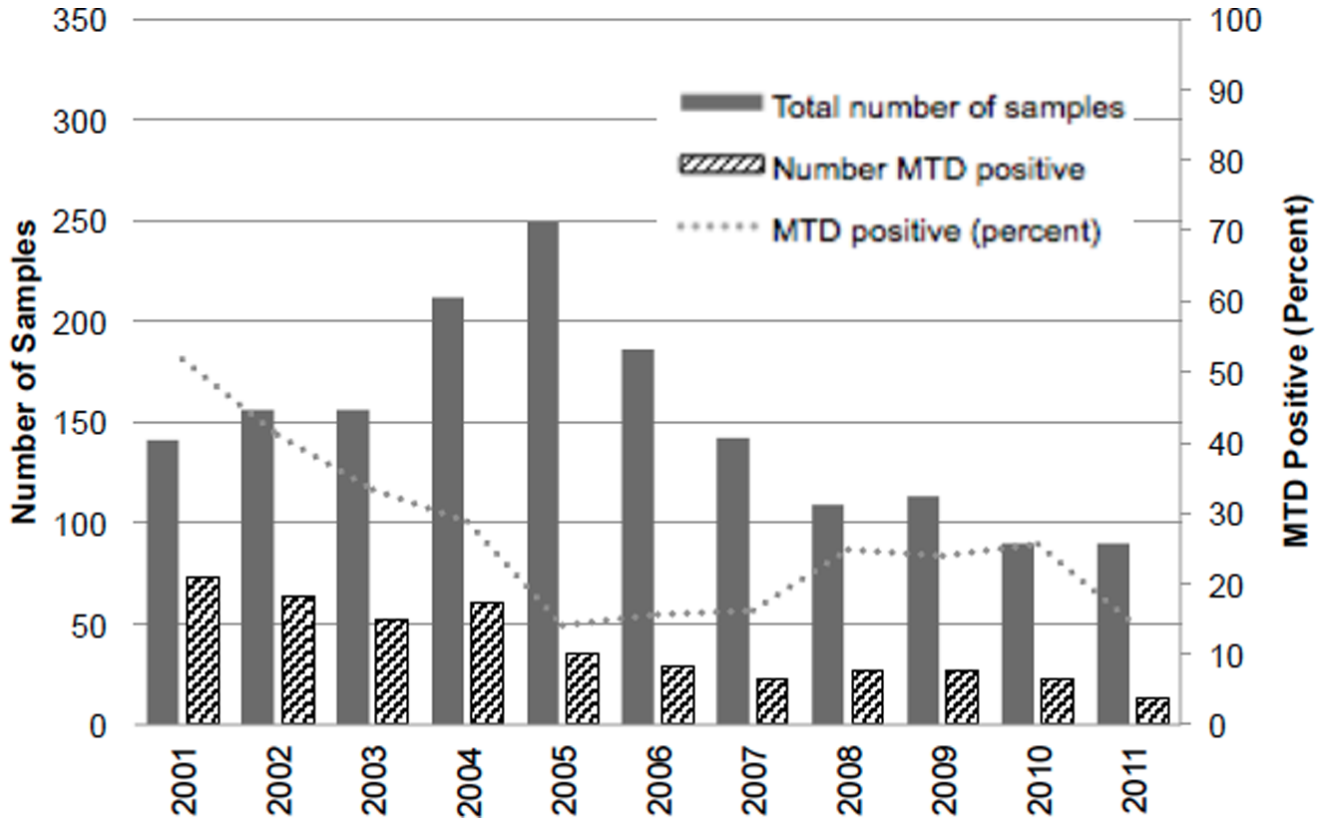
Trend in the number of samples submitted for MTD testing, number with positive MTD, and the percent of samples with positive MTD by year, per unique visit.

The trends in the numbers of MTD testing and the numbers and percentages of MTD-positive samples during the study period are shown in Figure 1 (per specimen) and Figure 2 (per unique visit). There were statistically significant downward trends in the number of MTD tests performed and the percentage of MTD positive samples during the study period (p<0.01 for both, data not shown). The absolute number as well as the proportion of MTD positive specimens were highest in 2001 (126, 57.8% per specimen; 73, 51.8% per unique visit, data not shown), whereas the total number of smear- positive specimen tested for MTD was the highest in 2005 (311 and 249 respectively, data not shown). The results per specimen and per unique visit were compared to see if the peak in 2005 was due to multiple MTD testing from the same visit or if there were simply more smear-positive specimens eligible for MTD, and the fact that the trends were similar suggests that the peak in 2005 is likely due to the increase in the total number of smear-positive specimens submitted for MTD compared to other years.

### Culture and Patient Characteristics

Culture and patient characteristics are summarized in Table 3 (per specimen) and in Table 4 (per unique visit). The majority of specimens came from males (male-to-female ratio 2.6 for both per specimen and per unique visit) and were sputum samples (94.9% per specimen, 95.3% per unique visit). The culture positive rates for TB were 28.5% of per specimen samples and 25.1% of per unique visit samples, respectively The culture positive rates for NTM were56.7% and 58.4%, respectively, and *Mycobacterium avium* complex (MAC) was most frequently isolated (46.3% and 47.3%, respectively). 14.1% of the samples per specimen and 16.5% of the samples per unique visit were culture negative.

**Table 3:** Patient demographics and summary of microbiological data of the specimens, per specimen.

	Total	HIV positive	HIV negative	Subtotal*	P value**
No. of specimen	2,240	1,431 (70.6%)	596 (29.4%)	2,027	
Median age	44 (13– 97)	43 (18–76)	48 (13–83)		<0.01
No. of specimen with known gender (M/F, ratio)	2,228 (1,611/617, 2.6)	1425 (1,032/393, 2.6)	595 (445/150, 3)	2,020 (1,477/543, 2.7)	0.27
Types of Respiratory Specimen					
Sputum (%)	2,125 (94.9)	1,364 (95.3%)	566 (95.0%)	1,930	0.77
Bronchial wash (%)	105 (4.7)	62 (4.5%)	27 (4.5%)	89	
Tracheal aspirate (%)	10 (0.5)	5 (0.4%)	3 (0.5%)	8	
Culture positive for TB (%)	639^1^(28.5)	233^1^ (16.3%)	309^1^ (51.8%)	542	<0.01
Culture positive for NTM	1,269 (56.7%)	969 (67.7%)	211 (35.4%)	1,180 (58.2%)	<0.01
Number of NTM (%)					
*Mycobacterium avium complex*	1,036^2^ (46.3%)	756^2^ (52.8%)	179 (30.0%)	935	<0.01
*Mycobacterium kansasii*	215^3^ (9.6%)	200^3^ (14.0%)	12 (2.0%)	212	<0.01
*Mycobacterium gordonae*	32^4^ (1.4%)	16^4^ (1.1%)	15 (2.5%)	31	0.02
*Mycobacterium xenopi*	5 (0.22%)	5 (0.3%)	0	5	
*Mycobacterium chelonae*-abscessus complex	4 (0.18%)	1 (0.1%)	3 (0.5%)	4	
Other mycobacteria	8 (0.36%)	4 (0.3%)	2 (0.3%)	6	
*Nocardia* spp.	3^5^ (0.13%)	2^5^ (0.1%)	0	2	
*Rhodococcus* spp.	4 (0.18%)	4 (0.3%)	0	4	
Culture Negative	315 (14.1%)	215 (15%)	74 (12.4%)	289	0.13

* Subtotal for those with known HIV status.

** p value between HIV positive and HIV negative.

1 17 out of 639 specimen was also culture positive for MAC; 7 in HIV positive group and 9 in HIV negative group.

2 4 were also culture positive for *M. gordonae*, 10 with *M. kansasii*, 2 with Nocardia. All were found among HIV positive group.

3 10 were also culture positive for MAC. All were from HIV positive group.

4 4 were also culture positive for MAC>All were from HIV positive group.

5 2 were also culture positive for MAC. All were from HIV positive group.

**Table 4:** Patient demographics and summary of microbiological data of the specimens, per unique visit.

	Total	HIV positive	HIV negative	Subtotal*	P value**
No. of specimen	1,644	1,058 (71.1%)	429 (28.9%)	1,487	
Median age	44 (13–97)	43 (18–76)	48.5 (13–83)		<0.01
No. of specimen with known gender (M/F, ratio)	1,633 (1,176/457, 2.6)	1,052 (751/301, 2.5)	428 (323/105, 3.1)	1,480 (1,074/406, 2.7)	0.11
Types of Respiratory Specimen (%)					
Sputum (%)	1,567 (95.3%)	1,017 (96.1%)	409 (95.3%)	1,426	0.48
Bronchial wash	72 (4.4%)	38 (3.6%)	19 (4.4%)	57	
Tracheal aspirate	5 (0.30%)	3 (0.3%)	1 (0.2%)	4	
Culture positive for TB	412^1^ (25.1%)	146^1^ (13.8%)	202^1^ (47.1%)	348	<0.01
Number of NTM (%)					
NTM	960 (58.4%)	721 (68.1%)	161 (37.5%)	882	<0.01
*Mycobacterium avium* complex	777^2^ (47.3%)	581^2^ (54.9%)	141 (32.9%)	722	<0.01
*Mycobacterium kansasii*	133^3^ (8.1%)	130^3^ (12.3%)	7 (1.6%)	137	<0.01
*Mycobacterium gordonae*	19^4^ (1.2%)	12^4^ (1.1%)	9 (2.1%)	21	0.14
*Mycobacterium xenopi*	2 (0.12%)	2 (0.2%)	0	2	
*Mycobacterium chelonae*	2 (0.12%)	0	2 (0.5%)	2	
Other mycobacteria	8 (0.49%)	5 (0.5%)	2 (0.5%)	7	
Nocardia spp.	2^5^ (0.12%)	1^5^ (0.1%)	0	1	
Rhodococcus spp.	2 (0.12%)	2 (0.2%)	0	2	
Culture Negative	271 (16.5%)	182 (17.2%)	66 (15.4%)	248 (16.7%)	0.40

* Subtotal for those with known HIV status.

** p value between HIV positive and HIV negative.

1 8 out of 412 also culture positive for MAC; 3 from HIV positive group, and 5 from HIV negative group.

2 3 also positive for *M. gordonae*, 6 with *M. kansasii*, and 1 with Nocardia. All from HIV positive group.

3 6 also positive for MAC. All from HIV positive group.

4 3 also positive for MAC.

5 1 also positive for MAC.

There were 2,027 specimens (90.5%) in the per-specimen group and 1,487 specimens (90.5%) in the per-unique visit group that had known HIV status, and the majority of them were HIV positive (70.6% per specimen, 71.1% per unique visit) (Table 3, 4). The median age of the HIV positive group was younger than the HIV negative group (43 years vs. 48 years, p<0.01). Of note, the proportion of specimens positive for TB was significantly higher in those specimens from HIV negative individuals (16.3% vs. 51.8%, p<0.01 per specimen; 13.8% vs. 47.1%, p<0.01 per unique visit). On the other hand, NTM, in particular MAC was isolated more from HIV positive samples (67.7% vs. 35.4%, p<0.01 per specimen; 68.1% vs. 37.5%, p<0.01 per unique visit).

We then looked to see if there were any relationships between the CD4 counts and culture positivity for TB among those who were HIV positive with known CD4 counts. The results are summarized in Table 5 (per specimen) and in Table 6 (per unique visit). There were 1,383 (61.7%) specimens (per specimen) and 998 (60.7%) specimens (per unique visit), respectively, which were from HIV positive individuals with known CD4 counts. Among those with HIV but without TB in the per specimen group, the median CD4 count was 34 cells/µL (range 0–1361). On the other hand, among those with HIV and TB, the median CD4 count was higher, being 82 cells/µL (range 1–624; p<0.01). The results were similar when reviewed per unique visit.

**Table 5:** Correlation between CD4 count and culture positivity for TB, per specimen (n = 1,383*).

	Total (n = 1,383)	TB positive (n = 221)	Non-TB** (n = 1162)	P-value
Median CD4 count (range)	38 (0–1361)	82 (1–624)	34 (0–1361)	P<0.01
Number CD4 <200 cells/µL (%)	1,131 (81.8%)	178 (80.5%)	953 (82.0%)	P = 0.6

* Number among those who are HIV positive with known CD4 counts.

**Table 6:** Correlation between CD4 count and culture positivity for TB, per unique visit (n = 998*).

	Total (n = 998)	TB positive (n = 134)	Non-TB** (n = 864)	P-value
Median CD4 count (range)	37 (0–1361)	82.5 (1– 446)	33 (0–1361)	P<0.01
Number CD4 <200 cells/µL (%)	828 (83.0)	108 (80.6%)	720 (83.3%)	P = 0.47

* Number among those who are HIV positive with known CD4 counts.

** Includes all results that were not TB.

## Discussion

Multiple studies have already shown the high yield of MTD in diagnosing TB especially in AFB smear-positive respiratory specimens [15]–[19]. To our knowledge, our study provides a summary of the largest number of AFB-smear positive respiratory specimens from a single institution with the MTD test. In the United States, both TB cases and rates have been decreasing steadily since the resurgence peak in 1992 [20], and similar trends have been observed in the state of Georgia as well [3]. At GMH also, the reported cases of TB have been steadily decreasing during the study period, reaching the lowest in 2011 (GMH, unpublished data). This is also reflected in the downward trend of the total number as well as the proportion of MTD-positive specimens during the study period (Figures 1, 2). In other words, the majority of AFB-positive samples submitted for MTD are positive for NTM, especially if the specimen came from a HIV-positive individual (Tables 3, 4). When looking at the total number of MTD tested, there is a peak in the number tested in 2005, which was observed both in the results per sample and per unique visit. Despite the increase, the total number of MTD-positive samples did not increase. This indicates that number of MTD tests performed in 2005 was mainly due to increased numbers of sputum test screenings and not due to an increased number of TB cases that year. It is possible that there was a scale-up of TB screening at that time; another possibility is that it took a while for routine MTD testing to be fully implemented, as the proportion of MTD positive samples seem to be relatively steady after 2005, around 20–30% (Figures 1, 2).

In our study, the majority of the samples were derived from HIV positive individuals, with more than 70% coming from HIV-positives among those with known status. In particular, those who were immunedeficient (CD4<200 cells/µL) constituted more than 80% of HIV positive individuals with known CD4 counts. The disproportionately high AIDS population in our study population is likely related to the enhanced isolation policy in place at GMH, which includes isolation of all HIV patients with respiratory symptoms or with abnormal chest radiographs. As patients with lower CD4 counts are more likely to develop pulmonary infections [21], they are more likely to be tested for TB with respiratory AFB. In addition, HIV patients are more likely to have their respiratory tract colonized with NTM [10]. It is therefore understandable why the proportion of TB was higher among the HIV negative group compared to the HIV positive group (Tables 3, 4). Given that advanced HIV is associated with increased risk of development of tuberculosis [22], [23], it is interesting to observe that the median CD4 count was higher among those who were positive for TB compared to those who were non-TB among our HIV positive group (Tables 5, 6). While the exact reasons are unclear, there are several possibilities to explain this: since those who have advanced immunodeficiency are more likely to have smear-negative TB [9] and since in principle, MTD was performed only on smear-positive samples, it is possible that smear-negative TB in the severely immunedeficient cases were not captured; another possibility is that a lower CD4 count is associated with increased pulmonary infection [21] and the non-TB patients among our HIV positive patients simply represent those with especially low CD4 counts who are more susceptible to other pulmonary infections.

One limitation of this study is that complete clinical information was not available for every patient; therefore, we could not ascertain all the clinical decisions by the clinicians. It is possible that some culture-negative TB cases have been missed from the analysis. Regardless, our study was able to show the utility of the NAAT over an 11-year period in a population that is characterized by a relatively high proportion of NTM among smear-positive samples, especially if the specimen came from a HIV-positive individual.

There are several studies that looked at the cost-effectiveness of NAAT in TB diagnosis, which suggested that NAAT may not be cost-effective in low-TB prevalence settings [24], [25]. Dowdy et al. stated that cost-effectiveness of MTD is sensitive to changes in the relative prevalence of TB among smear-positive patients, annual number of specimens processed by the laboratory, and the marginal cost of reagents [24]. More recently, a study by Marks et al showed that MTD was cost effective in certain high-risk populations including HIV [19]. Given that our patient population is characterized by the high proportion of NTM, especially in HIV-positives that comprised the majority of our study population, using the test on a routine basis in our setting is likely to be cost-effective.

After the new Xpert MTB/RIF NAAT assay received FDA approval in July 2013 [26], we started to offer the test in the same way as MTD on smear positive respiratory samples. Xpert MTD/RIF has several advantages over MTD. For example, while MTD is a manual test, Xpert MTD/RIF is a self-contained automated test, with less potential for PCR contamination. In addition, the platform can be used to detect other pathogens. Teo et al. [27] reported that the overall testing characteristics of Xpert MTD/RIF and MTD were comparable, whereas MTD resulted in a higher proportion of equivocal results compared to Xpert MTD/RIF (10.5% vs. 5.5%). The proportion of equivocal results was much smaller (1.8%) in our study, and the differences may be because we only looked at smear-positive respiratory samples, whereas Teo et al. looked at both respiratory and non-respiratory samples [27]. Additional studies are needed to assess the true cost-effectiveness and the processing capacity, especially in a setting with a relatively high volume of samples.

## Summary and Conclusion

Our study showed excellent clinical performance and utility of NAAT for diagnosis of TB from AFB smear-positive samples. This is especially significant in a clinical setting that is characterized by a high-proportion of immunodeficienct HIV-positive individuals, who are more likely to have NTM rather than TB. It is thus appropriate to utilize NAAT to diagnose or rule out TB in populations with a high prevalence of HIV.
